# Cognition among individuals along a spectrum of increased risk for Parkinson’s disease

**DOI:** 10.1371/journal.pone.0201964

**Published:** 2018-08-20

**Authors:** Lana M. Chahine, Liz Urbe, Chelsea Caspell-Garcia, Dag Aarsland, Roy Alcalay, Paolo Barone, David Burn, Alberto J. Espay, Jamie L. Hamilton, Keith A. Hawkins, Shirley Lasch, James B. Leverenz, Irene Litvan, Irene Richard, Andrew Siderowf, Christopher S. Coffey, Tanya Simuni, Daniel Weintraub, the Parkinson’s Progression Markers Initiative

**Affiliations:** 1 University of Pittsburgh, Pittsburgh, PA, United States of America; 2 The University of Iowa, Iowa City, Iowa, United States of America; 3 Department of Old Age Psychiatry, Institute of Psychiatry, Psychology & Neuroscience, King’s College London, England; 4 Columbia University Medical Center, Department of Neurology, New York, NY, United States of America; 5 Department of Medicine and Surgery, Center for Neurodegenerative Diseases, University of Salerno, Fisciano, Italy; 6 Institute for Ageing and Health, Newcastle University, Newcastle, United Kingdom; 7 Department of Neurology, University of Cincinnati Academic Health Center, Cincinnati, OH, United States of America; 8 The Michael J. Fox Foundation for Parkinson’s Research, New York, NY, United States of America; 9 Department of Psychiatry, Yale School of Medicine, New Haven, CT, United States of America; 10 Institute for Neurodegenerative Disorders, New Haven, CT, United States of America; 11 Cleveland Clinic, Cleveland, OH, United States of America; 12 UCSD Movement Disorder Center, Department of Neurosciences, University of California San Diego, San Diego, CA, United States of America; 13 Departments of Neurology and Psychiatry, University of Rochester School of Medicine and Dentistry, Rochester, NY, United States of America; 14 Department of Neurology, University of Pennsylvania Perelman School of Medicine, Philadelphia, PA, United States of America; 15 Northwestern University Feinberg School of Medicine, Chicago, IL, United States of America; 16 Department of Psychiatry, University of Pennsylvania School of Medicine, Philadelphia, PA, United States of America; 17 Parkinson’s Disease and Mental Illness Research, Education and Clinical Centers (PADRECC and MIRECC), Philadelphia Veterans Affairs Medical Center, Philadelphia, PA, United States of America; Oslo Universitetssykehus, NORWAY

## Abstract

**Introduction:**

Several characteristics associated with increased risk for Parkinson’s disease (PD) have been identified, including specific genotypes and various non-motor symptoms. Characterizing non-motor features, such as cognitive abilities, among individuals considered at-risk for PD is essential to improving prediction of future neurodegeneration.

**Methods:**

Participants belonging to the following cohorts of the Parkinson Progression Markers Initiative (PPMI) study were included: de novo PD with dopamine transporter binding deficit (n = 423), idiopathic REM sleep behavior disorder (RBD, n = 39), hyposmia (n = 26) and non-PD mutation carrier (NMC; Leucine-rich repeat kinase 2 (LRRK2) G2019S (n = 88) and glucocerebrosidase (GBA) gene (n = 38) mutations)). Inclusion criteria enriched the RBD and hyposmia cohorts, but not the NMC cohort, with individuals with dopamine transporter binding deficit. Baseline neuropsychological performance was compared, and analyses were adjusted for age, sex, education, and depression.

**Results:**

The RBD cohort performed significantly worse than the hyposmia and NMC cohorts on Symbol Digit Modality Test (mean (SD) 32.4 (9.16) vs. 41.8 (9.98), p = 0.002 and vs. 45.2 (10.9), p<0.001) and Judgment of Line Orientation (11.3 (2.36) vs.12.9 (1.87), p = 0.004 and vs. 12.9 (1.87), p<0.001). The RBD cohort also performed worse than the hyposmia cohort on the Montreal Cognitive Assessment (25.5 (4.13) vs. 27.3 (1.71), p = 0.02). Hyposmics did not differ from PD or NMC cohorts on any cognitive test score.

**Conclusion:**

Among individuals across a spectrum of risk for PD, cognitive function is worse among those with the characteristic most strongly associated with future risk of PD or dementia with Lewy bodies, namely RBD.

## Introduction

In Parkinson’s disease (PD), the second most common neurodegenerative disorder, motor symptoms constitute the core diagnostic criteria[1]. However, the pathophysiological changes of PD begin years to decades before clear-cut motor symptoms manifest[2]. These manifestations include a cluster of at-risk characteristics or prodromal manifestations. Therefore, the definition of PD has been extended to include individuals considered at-risk for PD. These fall along a broad spectrum of risk: asymptomatic carriers of mutations associated with PD, as well as individuals with prodromal non-motor clinical signs/symptoms, biomarker findings, or genetic polymorphisms that alone or in combination predict increased risk for PD to varying degrees[3].

In many cases among individuals at-risk for PD, the course/progression to the motor manifestations of PD aligns well, both anatomically and temporally, with the neuropathological staging system proposed by Braak[4,5], as follows: (1) In Braak stage I, involvement of the olfactory tubercle and medulla manifests clinically with hyposmia (i.e., impaired olfaction), reduced heart rate variability, and other manifestations of autonomic dysfunction; (2) In Braak stage II, there is involvement of more rostral brainstem structures, including the serotonergic dorsal raphe nuclei, which clinically may manifest with anxiety and depression, and the glutamatergic peri-locus coeruleus, which has been hypothesized to lead to REM sleep behavior disorder [RBD]. Involvement of norepinephrine-producing neurons in the locus coeruleus at this stage may also mediate subtle abnormalities in cognition [e.g., attention and working memory] reported in the prodromal PD state[6].

Data on cognition in individuals at-risk for PD are limited, and cognitive changes in subgroups across the at-risk spectrum have not been well described. In addition to enrolling a cohort of de novo PD patients, the Parkinson Progression Markers Initiative (PPMI) study also enrolled individuals without a diagnosis of PD but who are considered at-risk for PD based on the presence of one of the following characteristics: genetic profile (i.e., carriers of Leucine-rich repeat kinase 2 (*LRRK2*) G2019S or glucocerebrosidase (*GBA*) gene mutations), hyposmia, or a diagnosis of RBD. This cohort thus represents a mixture of individuals, some who are at-risk for PD but who will never develop it, as well as individuals that may be in the PD prodrome, presumed to be manifesting the earliest signs of neurodegeneration. For brevity, in this manuscript this cohort will hereto forth be referred to as the “at-risk PPMI cohort”. The at-risk PPMI cohort provides a unique opportunity to investigate differences in cognition among at-risk subgroups. Based on Braak staging, we hypothesized a “gradient of prodromalness” in which the RBD cohort would have worse cognition than the hyposmia cohort, which in turn would have worse cognition than the non-PD mutation carrier (NMC cohort). In this study, we investigated this hypothesis in the at-risk PPMI cohort.

## Methods

### Study participants

PPMI is a multicenter, international, longitudinal cohort study. Study aims, methodology, and details of study assessments have been published elsewhere[7] and are available on the PPMI website (http://www.ppmi-info.org/study-design). PPMI includes several study cohorts. Inclusion criteria vary based on the cohort, as detailed below. Exclusion criteria applying to all cohorts included in this analysis were: (i) dementia based on the site investigator’s clinical assessment and (ii) any medical conditions precluding participation at the discretion of the investigator.

PPMI includes 4 cohorts of participants included in this analysis:

1. PD cohort (n = 423): newly diagnosed, untreated at enrollment. PD patients were required, at baseline, to have been diagnosed within two years of study enrollment, have dopamine transporter (DAT) binding deficit based on visual interpretation of DaTscan SPECT (as described in the supporting information), and be untreated for PD.
2. RBD cohort (n = 39). RBD was diagnosed by the site principal investigator (based on clinical history along with polysomnographic findings, where available). Exclusion criteria for this cohort included motor signs that meet criteria for a diagnosable parkinsonian syndrome based on the opinion of the investigator. In order to enrich this cohort with individuals presumed to have incipient motor PD[8], they underwent DAT imaging. All those who had DAT binding deficit (as defined in S1 File) qualified for inclusion in PPMI. In addition, approximately 10% of those without a DAT binding deficit were also included, with the goal of keeping site investigators blinded to DAT SPECT results.
3. Hyposmia cohort (n = 26). Olfaction was measured using the University of Pennsylvania Smell Identification Test (UPSIT)[9]. Any individual without a diagnosis of PD was eligible to undergo olfactory testing. Recruitment for this cohort occurred from various sources including the community (via targeted online ads) and PPMI sites’ outpatient clinics. Individuals expressing interest in olfactory testing were mailed an UPSIT, and they mailed completed UPSITs back to a central “olfaction core” which scored the smell tests and contacted individuals meeting criteria for hyposmia. Hyposmia was defined as a score of <10^th^ percentile for age and sex. These individuals were then seen at a PPMI site for a screening visit. In order to enrich this cohort with individuals presumed to have incipient motor PD[10–12]) a DAT SPECT was performed at screening. All those who had a DAT binding deficit qualified for inclusion in PPMI. In addition, approximately 10% of those without a DAT binding deficit were also included, with the goal of keeping site investigators blinded to DAT SPECT results.
4. Non-manifesting mutation carrier (NMC) cohort (n = 126). These were individuals without a diagnosis of PD who are carriers of the G2019S mutation in the *LRRK2* gene (n = 88), or the following *GBA* mutations (n = 38): 84GG (c.115+1G>A), IVS2+1G>A, c.1226A>G (N370S), c.1448T>C (L444P). These individuals were identified through various sources. For example, any adult who was Ashkenazi Jewish and had a 1st degree relative with PD could be referred for telephone-based genetic counseling and screened for the LRRK2 G2019S and GBA mutation, or individuals with a known mutation (regardless of how it was identified) could have self-referred for participation. PPMI also enrolled carriers of synuclein (SCNA) gene mutations but given the small number enrolled at the time of this analysis (n = 5) this subgroup was not included.

The study protocol was approved by the institutional review board of the University of Rochester. Institution review board approval was also obtained at each PPMI site. Written informed consent was obtained from all study participants.

### Assessments

Assessments obtained on the PPMI cohort and considered in these analyses included:

- Demographics and handedness: age at baseline, sex, education, and self-reported handedness (because only 2% of the cohort reported mixed handedness these were combined with the right-handed group).
- Neuropsychological test battery (the domains tested by the respective test is indicated, preceding the test name): Global cognitive function—Montreal Cognitive Assessment (MoCA)[13], Processing speed/attention—Symbol Digit Modalities Test (SDMT)[14], Executive function/working memory—Semantic fluency[15] (number of words generated for animals, vegetables, fruit) and Letter-Number Sequencing (LNS), Verbal memory—Hopkins Verbal Learning Test-Revised (HVLT-R)[16], immediate and delayed free recall and recognition discrimination, Visuospatial function—Benton Judgment of Line Orientation (JOLO) 15-item (split-half) version[17].

Participants were categorized as having mild cognitive impairment (MCI) if they scored >1.5 SD below the mean on ≥2 detailed neuropsychological test scores, regardless of cognitive domain [18].

- Depression assessment—15-item Geriatric Depression Scale (GDS-15) [19]
- DAT SPECT—DAT SPECT was performed as previously described[7]. A binary determination of DAT binding deficit was made in the at-risk cohort based on the definition described in supplementary material. The striatal specific binding ratio (SBR) was also considered.
- Olfaction—UPSIT scores were used to categorize all participants into olfactory levels of normosmia, hyposmia, and anosmia based on age and sex-specific normative values[9,20],
- RBD—REM Sleep Behavior Disorder Questionnaire (RBDSQ)[21]. The cutoff score indicative of possible RBD was ≥6 in the PD cohort[22] and ≥5 in all other cohorts[21].

### Statistical analysis

All clinical and biomarker data included in this study were downloaded from the PPMI database on August 1, 2016. Baseline characteristics were summarized using descriptive statistics, and compared across cohorts using generalized linear models assuming a normal distribution for continuous variables and a binomial distribution for categorical variables.

Differences in variables of interest among the 4 cohorts were examined using generalized linear models for continuous variables and logistic regression models for categorical variables. The following variables were examined, each in a separate model: cognitive test scores, presence of MCI, UPSIT, presence of DAT binding reduction, DAT SSBR, and presence of possible RBD based on RBDSQ score. A normal distribution was assumed for continuous variables and a binomial distribution for categorical variables. Age, sex, education and GDS-15 score were included as co-variates. For any variables that showed a significant difference with a p-value = 0.1 or less, pairwise comparisons between all cohort combinations were performed, and values with p<0.05 were regarded as statistically significant.

A sub-group analysis, utilizing the same statistical tests, was performed comparing the GBA and LRRK2 mutation carriers that constitute the NMC cohort.

Adjustments for multiple comparisons were not made given the exploratory nature of this analysis.

Statistical analyses were performed using SAS 9.4 (SAS Institute Inc., Cary, NC).

## Results

Baseline demographic characteristics are shown in Table 1.

**Table 1:** Demographics, clinical, and DAT SPECT characteristics in the PD, RBD, Hyposmia, and non-PD mutation carrier groups.

Variable	PD cohort (N = 423)	RBD cohort (N = 39)	Hyposmia cohort (N = 26)	Non-PD mutation carriers (N = 126)	Asymptomatic LRRK2 mutation carriers (N = 88)	Asymptomatic GBA mutation carriers (N = 38)	p-value* for test of difference between groups)	p-value* for test of difference between LRRK2 and GBA groups only
**Age** Mean (SD; range)	61.6 (9.7; 33–85)	69.6 (5.5; 59–82)	68.1 (6.2; 61–83)	62.2 (7.3; 50–84)	61.6 (7.1; 50–81)	63.6 (7.5; 52–84)	< 0.0001	0.1578
Sex Male N (%):Female N (%)	277 (65): 146 (35)	33 (85): 6 (15)	18 (69): 8 (31)	83 (66):43 (34)	32 (36):56 (64)	11 (29):27(71)	< 0.0001	0.4214
**Education**								
< 13 yrs N(%): ≥13 years N (%)	76 (18):347 (82)	14 (36):25 (64)	3 (12): 23 (88)	26 (21):97 (77)	23 (26):63 (72)	3 (8):34 (89)	0.0433	0.0341
Number with Missing Data N (%)	0	0	0	3 (2)	2 (2)	1 (3)		
**Self-reported handedness**								
Right or Mixed N(%):Left N(%)	385 (91):38 (9)	39 (100):0 (0)	23 (88)3 (12)	104 (83): 18 (14)	73 (83): 12 (14)	31 (82): 6 (16)	0.1261	0.8729
Number with Missing Data N (%)	0	0	0	4 (3)	3 (3)	1 (3)		
**Geriatric Depression Scale-15**								
Mean (SD; range)	2.3 (2.4; 0–14)	2.8 (2.6;0–10)	1.5 (1.5; 0–6)	1.7 (2.1; 0–9)	1.6 (2.0)	1.9 (2.4; 0–9)	0.0063	0.4126
Number with Missing Data N (%)	0	0	0	6 (5)	5 (6)	1 (3)		
**UPSIT (categorical)**								
Normosmia N (%)	39 (9)	1 (3)	0 (0)	44 (35)	27 (31)	17 (45)		
Hyposmia N (%)	237 (56)	18 (46)	7 (27)	80 (63)	57 (65)	18 (47)	< 0.0001	0.1722
Anosmia N (%)	147 (35)	18 (46)	19 (73)	3 (2)	2 (2)	1 (3)		
Number with Missing Data N (%)	0 (0)	2 (5)	0 (0)	4 (3)	2 (2)	2 (5)		
REM sleep behavior disorder (score ≥ 5)**								
No N (%): Yes N (%)	312 (74): 108 (26)	4 (10): 34 (87)	15 (58): 11 (42)	92 (73): 23 (18)	67 (76):16 (18)	25 (66):7 (18)	< 0.0001	0.7104
Number with Missing Data N (%)	3 (1)	1 (3)	0	11 (9)	5 (6)	6 (16)		
**DAT binding deficit**								
No N (%):Yes N(%)	1 (0.2): 413 (98)	3 (8): 36 (92)	4 (15): 22 (85)	83 (66): 18 (14)	56 (64):16 (18)	27 (71):2 (5)	< 0.0001	0.0332
Number with Missing Data N (%)***	4 (1)	0	0	25 (20)	16 (18)	9 (24)		
**Mean striatal specific binding ratio**								
Mean (SD)	1.4 (0.40;0–3)	1.5 (0.39; 1–3)	1.9 (0.40; 1–3)	2.6 (0.50; 1–4)	2.5 (0.49’ 2–4)	2.7 (0.50; 1–4)	< 0.0001	0.0002
Number with Missing Data N (%)***	4 (1)	0	0	25 (20)	16 (18)	9 (24)		

*Generalized linear models were used to test for differences in continuous variables and a logistic regression model was used to test for differences in categorical variables

** The cutoff score indicative of possible RBD was ≥6 in the PD cohort[21] and ≥5 in all other cohorts[20]. Note that the diagnosis of RBD in the RBD group was based on interview and not necessarily RBDSQ score. Furthermore, it is likely the majority of individuals with RBD in the RBD group were being treated at the time of enrollment in PPMI/completion of this questionnaire.

***1 subject was enrolled but terminated study participation prior to undergoing DaTscan. 3 subjects were enrolled at sites in a country in which DaTscan is not available. These participants underwent AV-133 imaging to determine their eligibility for study participation

Mean age, sex, education level, and GDS-15 were significantly different between at least two of the cohorts. As a result, all subsequent between-group analyses were adjusted for age, sex, education and GDS-15 scores.

Olfaction was significantly more impaired, and RBDSQ score higher, in the PD, RBD, and hyposmia cohorts compared to the NMC cohort. As expected, most subjects in the PD, RBD, and hyposmia cohorts had a DAT binding deficit, whereas less than 20% of the NMC cohort had a DAT binding deficit. The mean striatal SBR was significantly lower in the PD cohort compared to all other cohorts (p<0.0001 for all pairwise comparisons). Mean striatal SBR was significantly lower in the RBD cohort compared to the hyposmia (p = 0.0009) and NMC (p<0.0001) cohorts, and the hyposmia cohort compared to the NMC cohort (p<0.0001).

Mean scores on the neuropsychological test battery in the four cohorts are shown in Table 2.

**Table 2:** Cognitive performance in the PD, RBD, hyposmic, and NPD-GC arms.

Cognitive Domain	Measure	PD Cohort (N = 423)	RBD Cohort (N = 39)	Hyposmic Cohort (N = 26)	Non-PD Mutation Carriers (N = 126)	p-value* for test of difference between groups)
**Mild cognitive impairment**	**2 or more tests > 1.5 SD below mean**					
	yes N(%): no N(%)	46 (10.9): 373 (88.2)	11 (28.2): 27 (69.2)	1 (3.8): 24 (92.3)	12 (9.5): 85 (67.5)	0.1477
	Number with Missing Data	4 (0.9%)	1 (2.6%)	1 (3.8%)	29 (23.0)	
**Global cognition**	**MoCA**					
	Mean (SD; range)	27.1 (2.32; 17–30)	25.5 (4.13; 11–30)	27.3 (1.71; 23–30)	26.9 (2.54; 19–30)	**0.0451**
	Number with Missing Data	3 (1)	0	0	3 (2)	
**Verbal memory**	**HVLT Immediate Recall**					
	Mean (SD)	24.4 (4.98; 9–36)	21.1 (5.12; 9–33)	22.8 (5.55; 12–33)	25.5 (5.89; 5–35)	0.3562
	Number with Missing Data N (%)	1 (0.2)	1 (3)	1 (4)	4 (3)	
	**HVLT Delayed Recall**					
	Mean (SD; range)	8.4 (2.52; 0–12)	6.5 (3.24; 0–12)	7.6 (3.37; 0–12)	9.1 (2.80; 0–12)	0.1496
	Number with Missing Data N (%)	1 (0.2)	1 (3)	1 (4)	4 (3)	
	**HVLT Delayed Recognition**					
	Mean (SD; range)	11.2 (1.23; 0–12)	10.5 (1.37; 7–12)	11.1 (1.39; 6–12)	11.2 (1.68; 0–12)	0.6388
	Number with Missing Data N (%)	2 (0.5)	1 (3)	1 (4)	7 (5)	
**Visuospatial function**	**Benton Judgment of Line Orientation**					
	Mean (SD; range)	12.8 (2.13; 5–15)	11.3 (2.36; 3–15)	12.9 (1.87; 8–15)	12.8 (2.05; 5–15)	**0.001**
	Number with Missing Data N (%)	1 (0.2)	2 (5)	1 (4)	4 (3)	
**Processing speed/attention**	**Symbol Digit Modalities Tes**t					
	Mean (SD; range)	41.2 (9.73; 7–82)	32.4 (9.16; 15–56)	41.8 (9.98; 16–55)	45.0 (10.9; 0–74)	**< 0.0001**
	Number with Missing Data N (%)	1 (0.2)	2 (5)	1 (4)	7 (5)	
**Executive function/working memory**	**Letter-Number Sequencing**					
	Mean (SD; range)	10.6 (2.66; 2–20)	9.0 (3.33; 3–17)	10.2 (1.80; 6–14)	10.7 (2.99; 2–20)	0.3796
	Number with Missing Data N (%)	1 (0.2)	1 (3)	1 (4)	4 (3)	
	**Semantic Fluency total**					
	Mean (SD; range)	48.7 (11.6; 20–103)	43.7 (8.74; 27–65)	47.0 (13.4; 26–75)	54.1 (13.8; 18–98)	**0.0084**
	Number with Missing Data N (%)	1 (0.2)	1 (3)	1 (4)	4 (3)	

*Analyses are adjusted for age, sex, education and GDS-15 score

**Fig 1:**
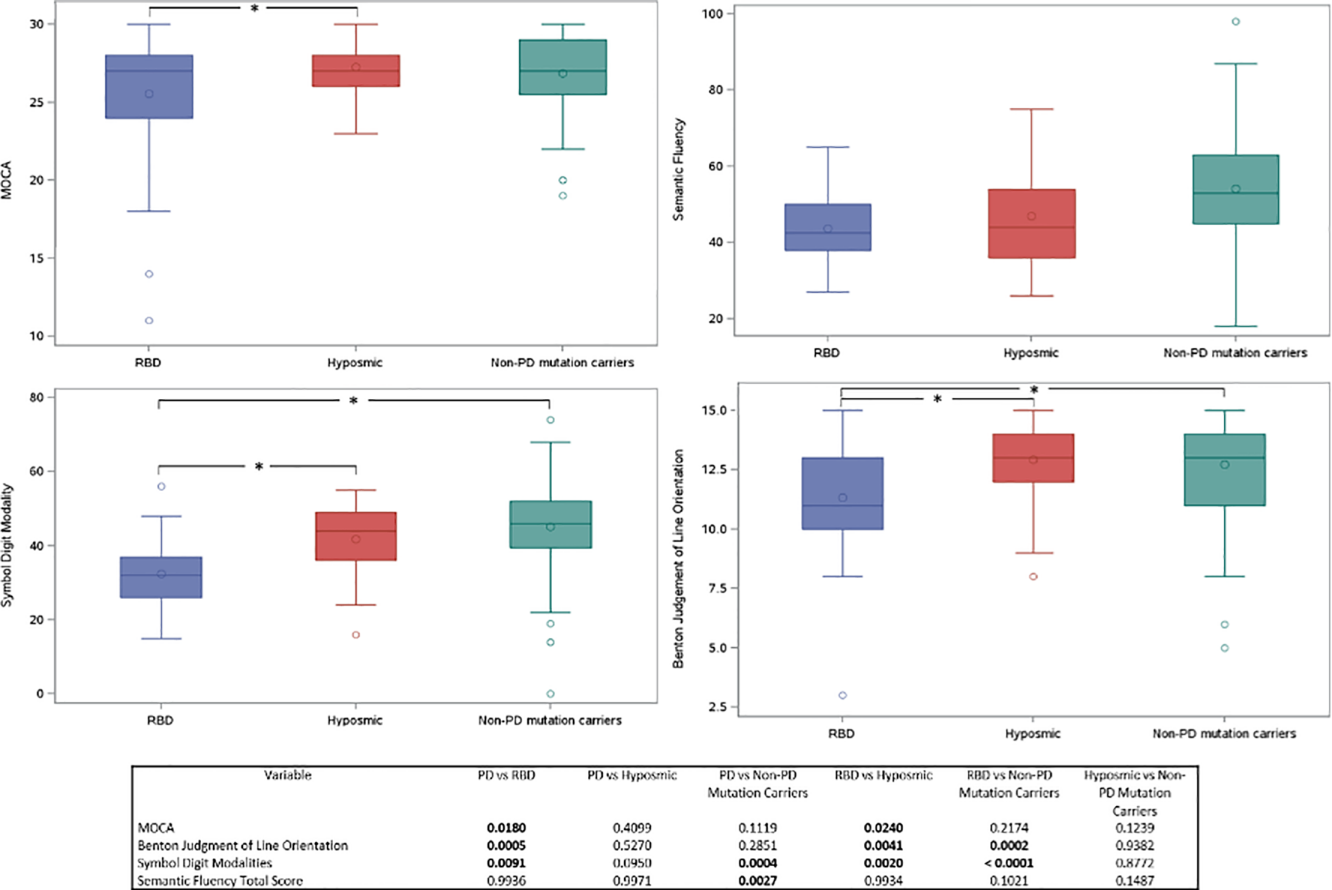
Graphical comparison of select neuropsychological test battery scores in the 3 at-risk groups. The scores for MoCA, semantic fluency, Symbol Digit Modalities Test (SDMT), and Benton Judgment of Line Orientation are shown for the 3 at-risk groups. Asterisks indicate significant difference in pairwise comparisons between groups.

P-values for pairwise comparisons between the different cohorts are shown in Fig 1. The measure of global cognition (MoCA score) was worse in the RBD cohort compared to the PD and hyposmia cohorts. The RBD cohort performed significantly worse on measures of two cognitive domains compared to all other cohorts: processing speed/attention (SDMT) and visuospatial function (JOLO) (Fig 1). Hyposmics did not differ from PD or NMC cohorts in any cognitive domain. The PD cohort performed significantly worse than the NMC on a measure of executive function (semantic fluency) and processing speed/attention (SDMT).

The RBD cohort had the highest prevalence of MCI compared to the other cohorts; however, none of the differences between groups was significant after adjusting for age, sex, education, and GDS-15 score.

In comparing the LRRK2 and GBA cohorts, the LRRK2 mutation carriers had lower scores on both global cognition (MoCA) and a measure of verbal memory (HVLT immediate free recall) (Table 3).

**Table 3:** Cognitive performance in the LRRK2 G2019S and GBA mutation carrier groups.

Cognitive Domain	Measure	Asymptomatic LRRK2 mutation carriers (N = 88)	Asymptomatic GBA mutation carriers (N = 38)	p-value* for test of difference between LRRK2 and GBA groups only
**Global cognition**	**MoCA**			
	Mean (SD; range)	26.5 (2.72; 19–30)	27.6 (1.86; 20–30)	**0.0427**
	Number with Missing Data N (%)	2 (2)	1 (3)	
**Verbal memory**	**HVLT Immediate Recall**			
	Mean (SD; range)	24.6 (6.03; 5–34)	27.5 (5.05; 14–35)	**0.0181**
	Number with Missing Data N (%)	2 (2)	2 (5)	
	**HVLT Delayed Recall**			
	Mean (SD; range)	8.8 (2.96; 0–12)	9.7 (2.27; 4–12)	0.1216
	Number with Missing Data N (%)	2 (2)	2 (5)	
	**HVLT Delayed Recognition**			
	Mean (SD; range)	11.1 (1.89; 0–12)	11.5 (0.93; 8–12)	0.3895
	Number with Missing Data N (%)	3 (3)	4 (11)	
**Visuospatial function**	**Benton Judgment of Line Orientation**			
	Mean (SD; range)	12.7 (2.08; 5–15)	12.9 (1.90; 8–15)	0.2660
	Number with Missing Data N (%)	2 (2)	2 (5)	
**Processing speed/attention**	**Symbol Digit Modalities Tes**t			
	Mean (SD; range)	44.2 (11.7; 0–74)	47.0 (8.49; 29–68)	0.3235
	Number with Missing Data N (%)	3 (3)	4 (11)	
**Executive function/working memory**	**Letter-Number Sequencing**			
	Mean (SD; range)	10.7 (3.14; 2–20)	10.9 (2.63; 6–18)	0.7698
	Number with Missing Data N (%)	2 (2)	2 (5)	
	**Semantic Fluency total**			
	Mean (SD; range)	54.0 (14.6; 18–98)	54.1 (11.5; 25–78)	0.7457
	Number with Missing Data N (%)	2 (2)	2 (5)	

*Analyses are adjusted for age, sex, education and GDS-15 score

## Discussion

In this study, we demonstrate significant differences in cognition among four cohorts presumed to be at-risk for PD, but to varying extents. As hypothesized, the RBD cohort performed worse than the other at-risk cohorts. RBD is thought to reflect a prodromal PD state resulting from neurodegeneration of pontine nuclei, including the glutamatergic peri-locus coeruleus. Involvement of nearby nuclei, including the noradrenergic locus coeruleus as well as the cholinergic pedunculopontine nucleus, could account for some of the cognitive dysfunction seen in RBD cases. Furthermore, the lower mean striatal SBR seen in this cohort compared to the hyposmia and NMC cohort indicates greater nigrostriatal dysfunction which could also help account for the worse cognition in this cohort[23].

Interestingly, and not consistent with our hypothesis, the RBD cohort was also more cognitively impaired than the PD cohort. The RBD cohort was predominantly older, male, and had a lower education level than other cohorts, all risk factors for cognitive impairment. It is likely that approximately half of the RBD cohort will develop dementia with Lewy Bodies [DLB][24] rather than idiopathic PD, in which cognitive dysfunction is mild early on[25]. This may partly explain the worse cognition in this cohort, possibly mediated by concomitant neurodegenerative disease pathology in the cortex and cholinergic nucleus basalis of Meynert, specifically Lewy body disease with or without Alzheimer’s disease pathology. The RBD cohort performed worse compared to all other cohorts in measures of processing speed/attention (SDMT) and visuospatial function (JOLO). This is of note considering that among individuals with RBD, abnormalities in tests of attention (as well as executive function) are predictive of future risk of DLB in RBD[26], and visuospatial dysfunction is a hallmark of DLB[27].

The hyposmia cohort did not differ from the PD cohort or the NMC cohort in any of the cognitive measures, despite significantly lower striatal SBRs. This is in contrast to the Parkinson Associated Risk Syndrome (PARS) cohort, in which individuals with both hyposmia and DAT binding reduction performed significantly worse on measures of global cognition, executive function/working memory, and verbal memory[6] compared to normosmics or hyposmics without DAT binding reduction. This discrepancy may be due to the small sample size (and reduced power) of the hyposmia cohort or to true intrinsic differences between the PPMI and PARS cohorts.

The NMC cohort includes predominantly healthy individuals, and the low prevalence of DAT binding reduction in that cohort suggests that at baseline they are indeed “low” on the spectrum of “prodromalness” (i.e., most of them have a low risk of conversion to motor PD). However, they are genetically heterogeneous and their risk of PD and its manifestations is likely largely influenced by their genotype. GBA mutations confer increased risk of cognitive dysfunction among individuals with PD[28], and this may result, pathophysiologically, from a synergistic effect between glucocerebrosidase dysfunction and alpha-synuclein pathology[29]. There are limited data on cognition in asymptomatic GBA mutation carriers. Similarly, there are limited data on cognition in asymptomatic LRRK2 G2019S carriers, but what data are available suggest that at least a subset of such individuals have worse performance on measures of executive function compared to non-carriers[30]. A study comparing cognitive function among asymptomatic GBA and LRRK2 mutation carriers found no differences between the cohorts[31]. In our cohort, while cognition was overall similar between the two cohorts, there were some differences. LRRK2 cohort participants had a lower mean MoCA and performed worse on a measure of verbal memory. Some of these findings may again be explained by evidence of greater nigrostriatal dysfunction[23] in the LRRK2 cohort. In addition, LRRK2 has higher penetrance for PD compared to GBA mutations [by age 85, estimates are approximately 30% for LRRK2[32] vs. 10% GBA mutations[33,34]]. Therefore, all other things being equal, a greater proportion of individuals at-risk for PD on the basis of LRRK2 mutations would be expected to have some degree of neuronal dysfunction or neurodegeneration [that could potentially manifest with cognitive dysfunction] compared to at-risk GBA mutation carriers.

In PD, GBA mutations associated with more severe phenotypes, such as L444P, are much more strongly associated with risk of dementia compared to other GBA mutations[35]. The sample size of the asymptomatic GBA cohort in PPMI limits genotype-phenotype correlations within this cohort at this time but will be of great interest as the sample size of this cohort increases (recruitment to this cohort is ongoing).

There are several limitations of this study, The Movement Disorders Society (MDS) research criteria for prodromal PD[3] were proposed after the at-risk cohort of PPMI was recruited and thus these criteria were not accounted for in the inclusion criteria. Rather, the at-risk PPMI cohorts were selected based on a range of at-risk or prodromal characteristics narrower than what the MDS criteria encompass, and the RBD cohort was also enriched for individuals with DAT binding deficit. These inclusion criteria likely limit the generalizability of our findings to other at-risk cohorts and the general population of individuals at-risk for PD. The latter, combined with the relatively small numbers in some of the cohorts, as well as missing data, limit conclusions that can be drawn, especially with respect to the hyposmia cohort. In addition, participants in all cohorts of the PPMI study may not be representative of the respective populations from which they are drawn. Comparison to individuals without known risk of PD was not possible as the healthy control cohort of PPMI was recruited with different exclusion criteria specifically in regards to cognition (i.e., individuals with a MoCA score of <27 were excluded from the healthy control cohort of PPMI, whereas this criterion was not applied to the other cohorts). Furthermore, the neuropsychological test battery, while relatively comprehensive in domain coverage, was limited in the number of tests used to examine each cognitive domain. In addition, some cohorts differed in global cognitive performance, and this alone may have influenced the differences in cognitive *profile* as well. Finally, while the administered cognitive tests preferentially represent specific cognitive domains, there is overlap in the cognitive domains measured, lowering the strength of the conclusions about affected cognitive domains.

Despite these limitations, our findings provide insight into the cognitive profile of individuals at-risk or in a prodromal state for PD. They lend support to the idea that there is a gradient of prodromalness that is consistent with the proposed Braak staging, such that individuals with manifestations presumably resulting from more rostral neurodegeneration, namely the RBD cohort, have worse cognition than hyposmics or asymptomatic carriers of PD-associated genes. Longitudinal follow-up of this cohort will yield additional insights across the spectrum of individuals at risk for PD and other neurodegenerative parkinsonian syndromes.

## Supporting information

S1 File Methods. (DOCX)
