# Supplementary material for: Cognition among individuals along a spectrum of increased risk for Parkinson’s disease
Source: PLoS One. 2018 Aug 20;13(8):e0201964. doi: 10.1371/journal.pone.0201964 (PMC6101368; doi:10.1371/journal.pone.0201964)
Supplement: S1 File — (DOCX) [file pone.0201964.s001.docx]

**S1 Methods**

**(see also ppmi-info.org)**

**DaTscan SPECT Visual Interpretation for the Parkinson’s Disease cohort**

DaTSCAN™ SPECT imaging was acquired at PPMI imaging centers per the PPMI imaging protocol and sent to the imaging core lab at the Institute for Neurodegenerative Disorders (IND) for visual interpretation. Visual assessment of screening DaTSCAN™ SPECT imaging for determination of dopamine transporter deficit was performed at IND by two expert readers in the field of nuclear medicine. Scans were read as either showing evidence of dopamine transporter deficit (i.e. abnormal) or not showing evidence of dopamine transporter deficit (i.e. normal.)

Normal images are characterized by two relatively symmetric comma or crescent-shaped focal regions of radiotracer uptake mirrored about the midline in transaxial images. Striatal uptake, comprising both the caudate and putamen, is distinctly evident compared to surrounding brain tissue. Abnormal images typically fall into at least one of the following three general categories: (i) Activity is asymmetric, e.g. uptake in the region of the putamen of one hemisphere is absent or greatly reduced with respect to the other. Uptake is still visible in the caudate nuclei of both hemispheres resulting in a comma or crescent shape in one and a circular or oval focus in the other. There may be reduced uptake between at least one striatum and surrounding tissues.

(ii) Ioflupane uptake is absent in the putamen of both hemispheres and confined to the

caudate nuclei. The signal is relatively symmetric and forms two roughly circular or

oval foci. Uptake of one or both is generally reduced. (iii) Uptake is absent in the putamen of both hemispheres and greatly reduced in one or both caudate nuclei. Uptake of the striata with respect to the background is reduced (see also DaTSCAN™ [package insert]. Arlington Heights, IL: GE Healthcare; 2011.)

**DaTscan SPECT Interpretation for the Prodromal Cohort**

DaTSCAN™ SPECT imaging was acquired at PPMI imaging centers per the PPMI imaging protocol and sent to IND for DAT eligibility. All scans were visually assessed and quantitatively analyzed to determine prodromal cohort enrollment for the PPMI study as described in the Method section below. All PPMI DAT scans were visually assessed by two different expert readers in the field of nuclear medicine for evidence of Dopamine transporter deficit. If a scan showed evidence of Dopamine transporter deficit as defined above, a score of "Positive" was assigned, otherwise, the scan was a scored as "Negative". Both visual assessment (VI) results were captured on the form as "VI Score 1" (reader 1 result) and "VI Score 2" (reader 2 result). The scans were also quantitatively analyzed via extracting count densities for the left caudate, right caudate, left putamen, right putamen, and occipital cortex and calculating striatal binding ratios (SBRs) for each of the 4 striatal regions. From the quantitative analysis the minimum putamen (calculated as the minimum SBR value from either the left and right putamen) and average putamen (calculated as the mean SBR value of the combined left and right putamen) were also determined. The minimum putamen value (*minput*) was then used, along with subject age at the time of the scan (*age*), to calculate a minimum putamen ratio which utilized the following formula:

$${minput}/{(-0.0153*age + 2.9576)}$$

The average putamen ratio was calculated similarly using the average putamen (*aveput*) and subject age at the time of the scan (*age*). This calculation utilized the following formula:

$${aveput}/{(-0.0161*age+3.1187)}$$

A prodromal cohort subject was deemed to have a DAT binding deficit eligible if at least one of the two VI Scores was positive and either the minimum putamen ratio OR average putamen ratio were less than 0.80. Additional expert review was performed on scans who did not meet the eligible criteria but fell in either of two categories:

- Two negative VI scores and either the minimum putamen ratio or average putamen ratio was less than or equal to 0.60
- Two positive VI scores and either the minimum putamen ratio or average putamen ratio was greater than or equal to 0.80.

If a scan did not meet the eligibility criteria or did not fall into either of the two categories for additional expert review, the scan was deemed not eligible.

80% of prodromal subjects with RBD, and/or hyposmia were to have striatal DAT binding <80% of that expected for based on the existing PPMI healthy subject database, and 20% with normal DAT binding were also included. Subjects and investigators were not informed the DAT binding this was to reduce investigator bias when evaluating longitudinal assessments for PD symptoms.
